# Interferon-Independent Innate Responses to Cytomegalovirus

**DOI:** 10.3389/fimmu.2019.02751

**Published:** 2019-12-11

**Authors:** Caroline L. Ashley, Allison Abendroth, Brian P. McSharry, Barry Slobedman

**Affiliations:** ^1^Discipline of Infectious Diseases and Immunology, Faculty of Medicine and Health, Charles Perkins Centre, University of Sydney, Camperdown, NSW, Australia; ^2^School of Microbiology, University College Cork, Cork, Ireland; ^3^APC Microbiome Ireland, University College Cork, Cork, Ireland

**Keywords:** interferon, cytomegalovirus, IFN-independent, ISG, herpes, innate immunity

## Abstract

The critical role of interferons (IFNs) in mediating the innate immune response to cytomegalovirus (CMV) infection is well established. However, in recent years the functional importance of the IFN-independent antiviral response has become clearer. IFN-independent, IFN regulatory factor 3 (IRF3)-dependent interferon-stimulated gene (ISG) regulation in the context of CMV infection was first documented 20 years ago. Since then several IFN-independent, IRF3-dependent ISGs have been characterized and found to be among the most influential in the innate response to CMV. These include virus inhibitory protein, endoplasmic reticulum-associated IFN-inducible (viperin), ISG15, members of the interferon inducible protein with tetratricopeptide repeats (IFIT) family, interferon-inducible transmembrane (IFITM) proteins and myxovirus resistance proteins A and B (MxA, MxB). IRF3-independent, IFN-independent activation of canonically IFN-dependent signaling pathways has also been documented, such as IFN-independent biphasic activation of signal transducer and activator of transcription 1 (STAT1) during infection of monocytes, differential roles of mitochondrial and peroxisomal mitochondrial antiviral-signaling protein (MAVS), and the ability of human CMV (HCMV) immediate early protein 1 (IE1) protein to reroute IL-6 signaling and activation of STAT1 and its associated ISGs. This review examines the role of identified IFN-independent ISGs in the antiviral response to CMV and describes pathways of IFN-independent innate immune response induction by CMV.

## Introduction

HCMV has a 236 kbp double stranded DNA (dsDNA) genome, 165 genes (1) encoding up to 751 protein products (2), a 45–100% seroprevalence in the adult population (3–7), and remains a significant human pathogen particularly in those with an underdeveloped or suppressed immune system. Just as HCMV infection can profoundly alter the overall adaptive immune response (8–13), it also generates a powerful innate response. Key mediators of this innate response are IFNs. There are three types of IFN: type I (α, β, κ, ω, τ, and ε), type II (γ), and type III (λ1, λ2, λ3, λ4). Type I and II IFNs are the best characterized in the context of HCMV and their induction, antiviral roles as well as the viral antagonism of these processes have been extensively reviewed (14–19). A role for type III IFNs, in the innate response to HCMV and murine CMV (MCMV), whose pathogenesis closely parallels that of HCMV (20), has recently been elucidated (21–27).

The innate response to both HCMV and MCMV infection is initiated when virus is detected by pattern recognition receptors (PRRs) including toll-like receptors (TLRs) TLR2 (28–31) and TLR9 (32–34). Once virus has bound and entered cells, HCMV and MCMV can be detected by cytosolic DNA sensors such as IFI16 (35, 36), ZBP1/DAI (37–39) and cGAS (32, 40) that signal through the stimulator of IFN genes (STING). Each of these pathways culminates in activation and dimerization of IRF3 resulting in production of type I IFN (41–44). Type I IFN production is subsequently enhanced by upregulation of IRF7, an ISG that is also capable of dimerizing and activating the type I IFN promoter (45). HCMV and MCMV infection both trigger production of type II IFN from CD8^+^ T cells, CD4^+^ T cells and natural killer (NK) cells (46–48). HCMV even remodels the IFNγ locus (IFNG) for sustained IFNγ expression in NKG2C^hi^ NK cells (49, 50). IFNλ production is induced by HCMV and MCMV infection (22) and these type III IFNs are themselves ISGs with production stimulated by IFNα and IFNβ treatment (51).

**Figure 1:**
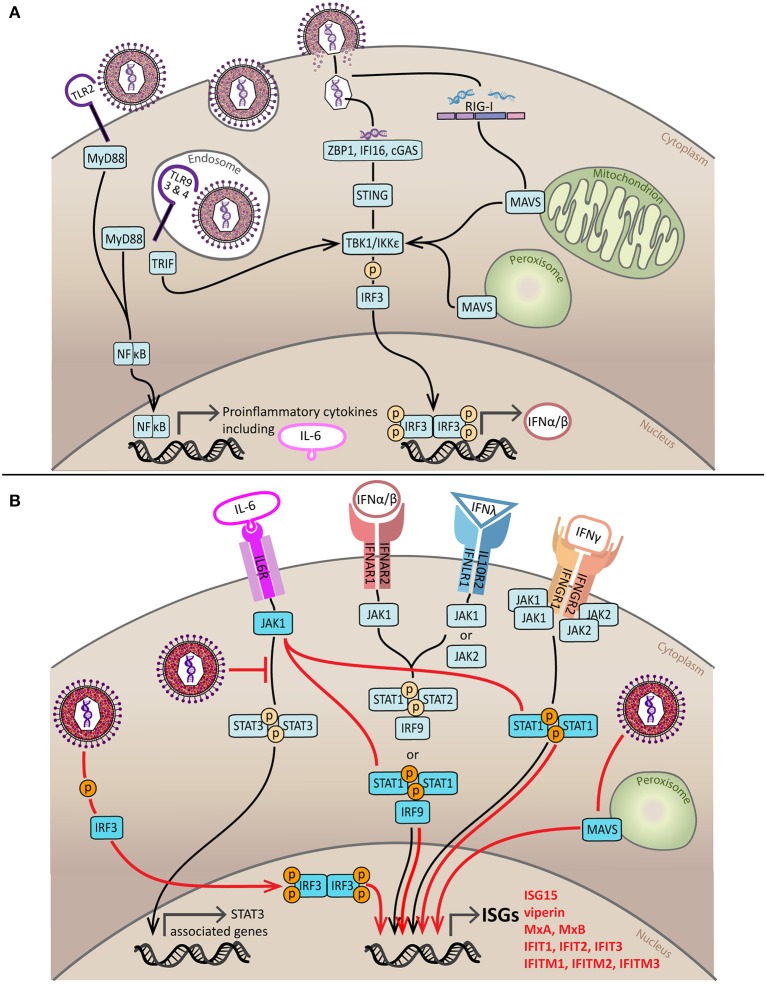
Induction and subversion of the innate IFN response by HCMV. **(A)** Sensing of HCMV by components of the innate immune response initiates production of IFNs and proinflammatory cytokines. HCMV is sensed by PRRs on the cell surface (TLR2) and in endosomes (TLR3, TLR4, and TLR9). Signaling from TLR2, TLR3, and TLR4 is through MyD88 and results in the activation and nuclear translocation NFκB, a transcription factor that stimulates expression of proinflammatory cytokines such as TNF, IL-8, IL-12, and IL-6. TLR9 and TLR4 signal through TRIF which causes activation by phosphorylation of IRF3 via TBK1/IKKε, activated IRF3 dimerizes and enters the nucleus to stimulate production of type I IFNs. HCMV infection can also be recognized by viral nucleic acid detectors in the cytoplasm; DNA sensors ZBP1, IFI16 and cGAS signal through ER-resident STING to activate TBK1/IKKε whilst the viral RNA sensor RIG-I activates TBK1/IKKε by signaling via MAVS located on the mitochondria or peroxisomes. The end result of both of these pathways is IRF3 phosphorylation, dimerization, nuclear translocation and production of type I IFNs. **(B)** IFN-dependent and IFN-independent pathways of ISG induction during HCMV infection. For IFN-dependent induction of ISGs to occur type I, type II and type III IFNs must bind to their cell surface receptors. Type I and III IFN receptors signal through various combinations of JAK proteins to phosphorylate STAT1 or STAT1 and STAT2 which form a complex referred to as ISGF3 with IRF9. ISGF3 then translocates to the nucleus where it binds to the ISRE to induce ISG production. The type II IFN receptor utilizes both JAK1 and JAK2 to phosphorylate STAT1, leading to its dimerization and nuclear translocation. Once in the nucleus, activated STAT1 dimers bind to GAS and stimulate ISG production. The three key pathways of HCMV-mediated IFN-independent ISG induction are indicated in red. Firstly, HCMV can directly activate IRF3; additionally, HCMV can sequester STAT3 and redirect the activated JAK1, created by IL-6 receptor binding, to phosphorylate STAT1; and finally peroxisomal MAVS may be able to trigger IFN-independent ISG expression at early times following infection. Black line = canonical IFN-dependent ISG induction pathway, red line = HCMV-induced, IFN-independent ISG induction pathway.

Key antiviral mediators of all IFN types are ISGs (52). Interferome, a database dedicated to chronicling all genes significantly regulated by IFN (changes ≥ 2-fold), identifies 12614 ISGs (53). Type I IFNs alone can trigger expression of more than 2,000 genes in humans, many of which are antiviral (54). Canonical induction of ISGs by type I, II, and III IFNs occurs by JAK/STAT signaling downstream of the type I IFN receptor (IFNAR1 + IFNAR2), the IFNγ receptor (IFNGR1 + IFNGR2) and the IFNλ receptor (IFNLR1 + IL10R2), respectively. The type I and II IFN receptors are widely expressed but type III IFN receptor expression is limited to epithelial cells (55, 56). ISGs stimulated by type I and III IFN contain an IFN stimulated response element (ISRE) in their promoter region that is bound by the activated transcription factor IFN stimulated gene factor 3 (ISGF3), comprised of phosphorylated STAT1 and STAT2 with IRF9 (55, 57–62), or by STAT2 homodimers associated with IRF9 (63–65). IFNγ induced ISG promoters contain γ-activated sequences (GAS) that are bound by STAT1 homodimers (66–70). However, upregulation of some ISG mRNAs in the early stages of HCMV infection (prior to DNA replication) are not inhibited by IFN neutralization (71, 72). Since this discovery, the body of literature demonstrating ISG induction independent of canonical IFN signaling pathways has been steadily expanding and those discussed in this review are summarized in Figure 1.

## IFN-Independent ISG Production

Initial differential display analyses compared the susceptibility of genes upregulated early vs. late in infection to inhibition by IFN neutralizing antibodies and/or protein synthesis inhibitor cyclohexamide (CHX) (72). Three of these genes: IFIT2/ISG54/p54/cig42, IFIT3/ISG60/p60/cig49 and viperin/cig6, were upregulated by HCMV at 8 h post infection (hpi) and even accumulated following exposure to replication-incompetent ultraviolent-irradiated HCMV (UV-HCMV) (72). Blocking type I IFN with neutralizing antibodies failed to inhibit IFIT2, and IFIT3 induction, demonstrating that their upregulation was both IFN-independent and could be triggered by viral binding entry alone (72). A subsequent, broad mRNA analysis using oligonucleotide arrays found that levels of 258 mRNAs were altered more than 4-fold prior to initiation of HCMV DNA replication (71). IFIT2 and IFIT3 were among these quickly detected ISGs as were MxA, MxB, and ISG15 (71). The immediacy of this induction suggests a direct mechanism requiring few intermediary steps, indeed IFIT2, IFIT3, ISG15 (73) and viperin (72) upregulation can be detected 6 hpi with HCMV in the absence of *de novo* host and viral protein synthesis (cyclohexamide (CHX) treatment). This is also the case for IFIT1/ISG56/p56 (73) and indicates that this subset of ISGs may be induced/upregulated independently of IFN during HCMV infection.

### IFN-Independent, IRF3-Dependent ISG Production

When searching for a mechanism underpinning IFN-independent ISG induction during CMV infection, initial studies turned to the powerful transcriptional regulator involved in IFN production, IRF3. Expression of constitutively active IRF3 in the absence of any viral stimulus could induce transcription of a subset of ISGs including IFIT1, IFIT2, IFIT3, ISG15, and viperin (74). IRF3-independent expression of these same ISGs was also observed during infection with other viruses: single stranded RNA (ssRNA) Newcastle disease virus (NDV) upregulated IFIT1, IFIT2 and ISG15 in cells that could respond to but were unable to produce type I IFN (75) and IFIT1 expression could be induced during ssRNA Sendai virus (SeV) infection by IRF3 nuclear translocation in cells unable to respond to type I IFN (76).

Studies using herpes simplex virus type 1 (HSV-1) demonstrated that IFIT1 expression could be driven by infection even in the presence of CHX in human fibroblasts (HFs) but could not be detected in the human epithelial osteosarcoma cell line U2OS (77). U2OS cells can respond to IFN but have defects in the STING signaling pathway (78) involved in IRF3 activation and dimerization in response to DNA sensing by IFI16, ZBP1/DAI, and cGAS (79–82). Furthermore, HSV-1 infection of IRF3^−/−^, IRF3^−/−^IRF9^−/−^, and IRF1^−/−^ murine fibroblasts revealed that IRF3 was essential for generation of an antiviral state and IFIT2 expression in response to UV-HSV-1 (83). In the case of IFIT1, expression was directly induced by an IRF3-containing complex binding to its promoter region (77, 84).

In the context of HCMV infection, initiation of IFIT2 transcription was found to occur independently of STAT1 nuclear localization (85) and in the presence of CHX (86). Soon it emerged that expression of IFIT1, IFIT2, IFIT3 and ISG15 during HCMV could be IFN-independent but always required IRF3 activation (42, 73, 87). Subsequent studies revealed that viperin expression could be driven directly by HCMV glycoprotein B (gB), in an IFN-independent, IRF3/IRF1 dependent manner (88, 89). This aligns with data demonstrating that IRF3 translocation to the nucleus is a requirement for the IFN-independent induction of an antiviral state in response to UV-HCMV (87). In contrast, another transcription factor implicated in type I IFN production NFκB (90), remains cytosolic (91).

To interrogate the IFN-independent, IRF3-dependent response to HCMV HFs have been engineered (92, 93) to lack either IRF3 through expression of the nPro protein of bovine viral diarrhea virus (BVDV) (nPro/HFs) which binds and degrades IRF3 (94) or STAT1, by expression of the parainfluenza virus type 5 (PIV-5) V protein (V/HFs) which targets STAT1 for proteasomal degradation (95). These nPro/HFs and V/HFs were recently utilized, alongside IRF3 KO CRISPR/Cas9 HFs, to demonstrate that expression of viperin, ISG15, IFIT1, IFIT2, IFIT3, Mx1, and Mx2 mRNA during infection with HCMV can be induced in an IRF3-dependent, STAT1-independent manner (96). In fact, mRNA levels of IFIT1, IFIT2, and IFIT3 were as highly elevated in the absence of STAT1-mediated IFNAR signaling as in the parental HFs (96) underlining the capacity of such IFN-independent mechanisms to profoundly regulate ISG expression. Many of these IFN-independent, IRF3-dependent ISGs are among the most potently induced by CMV infection and examining the roles these genes play in the innate response to CMV is essential to understanding the ramifications of this non-canonical regulation.

#### Viperin

Viperin inhibits the egress and replication of many viruses (97–102). However, in the context of HCMV, viperin upregulation is proviral, initiated by infection to manipulate cellular metabolism and cause the accumulation of cytosolic lipids for use in production of the viral envelope (103). In trophoblasts, a cell type of particular clinical relevance due to their role in the transmission of congenital HCMV (104–106), viperin is required for efficient expression of immediate early viral genes (107). Viperin is also known to enhance type I IFN production in plasmacytoid dendritic cells (pDCs) by localizing to lipid rafts and acting as a scaffold for recruitment of interleukin-1 receptor-associated kinase 1 (IRAK1) and TNF receptor associated factor 6 (TRAF6) (108).

In addition, viperin has been identified to act in its capacity as a member of the radical *S*-adenosyl-L-methionine (SAM) superfamily of enzymes to facilitate conversion of cytidine triphosphate (CTP) to 3′-deoxy-3′,4′-didehydro-CTP (ddhCTP) (109). Thus far, ddhCTP is known to act as a terminator of RNA synthesis by viral (Dengue and Zika) RNA-dependent RNA polymerases (109) and so investigations into its interaction with the HCMV encoded viral DNA polymerase are warranted. The viperin gene (RSAD2) lies in close proximity to the gene encoding cytidylate monophosphate kinase 2 (CMPK2) in the genome, suggesting a potential functional link to this pathway (109). Expression of CMPK2 is so closely linked to viperin that, following stimulation by IFN, viperin, CMPK2 and a long non-coding RNA (lncRNA) called lncRNA-CMPK2 are all co-transcribed (110). Interestingly, lncRNA-CMPK2 acts as a negative regulator of ISG expression (including ISG15, IFIT3 and IFITM1) (110). If IFN-independent, CMV-induced viperin upregulation also enhances expression of lncRNA-CMPK2, this could be a novel mechanism utilized by the virus to dampen the antiviral ISG response.

Furthermore, viperin has been demonstrated to be important for replication of Kaposi's sarcoma-associated herpesvirus (KSHV), a function attributed to the ability of viperin to catalyze oxidation of methionine in the viral DNA helicase, enhancing its expression and function (111). In this context, IFN-independent viperin upregulation by HCMV may be a way to ensure viral replication proceeds with maximum efficiency and thus the potential of viperin to modify the HCMV viral helicase-primase complex should be considered for further study. Overall, IFN-independent upregulation of viperin by HCMV seems to be a process initiated by the virus very early in infection to prepare the cell for its role as a virus-producing factory.

#### ISG15

ISG15 is a small ubiquitin-like protein that exists in three forms: (1) unconjugated within the cell, (2) conjugated within the cell (112, 113), and (3) secreted into serum (mainly by granulocytes) where it promotes NK maturation and IFNγ production (114). During HCMV infection accumulation of both free and conjugated ISG15 can be partially inhibited by interfering with the canonical IFNAR signaling pathway with a JAK inhibitor (115) but some IFN-independent, IRF3-dependent expression remains (96). Whilst the mechanisms by which ISG15 regulates CMV infection are currently unknown, it appears to possess antiviral activity as blocking ISG15 accumulation enhances viral replication (115) and HCMV antagonizes both the production of unconjugated ISG15 and ISGylation (115–118).

On the other hand, it is interesting to note that whilst in murine studies ISG15^−/−^ mice are generally more sensitive to disseminated viral infections (119) human patients presenting with primary immunodeficiencies associated with defects in ISG15 expression are not (120). In fact, ISG15^−/−^ fibroblasts isolated from such patients and primed with type I IFN were less susceptible to infection with HCMV than controls. This was attributed to the elevated levels of antiviral ISGs in these cells, a result of ISG15s ability to bind and stabilize the E3 ubiquitin ligase-like protein USP18, which acts as a negative regulator of the type I IFN response (120, 121).

It is also possible that HCMV manipulates levels of ISG15 to shift monocytes toward the mixed M1/M2 macrophage phenotype that is observed during infection (122) and hypothesized to enhance viral dissemination and persistence (123, 124). This is because in the absence of infection, ISG15 plays a role in the maintenance of mitochondrial homeostasis (125). Specifically, ISGylation of mitochondrial components can control mitochondrial function: reducing the rate of oxidative phosphorylation (OXPHOS) and causing a corresponding decrease in mitochondrial reactive oxygen species (ROS) (126). A reduction in levels of mitochondrial ROS alters macrophage polarization, shifting these cells toward a mixed M1/M2 phenotype (126).

#### IFITs

IFITs are ISGs with antiviral capabilities against flaviviruses, poxviruses, coronaviruses and papillomaviruses (127–130). A pan-viral mechanism of host defense mediated by IFITs is the sequestration of eukaryotic initiation factor (eIF3) by IFIT1 which slows the overall rate of cellular protein synthesis (76, 84, 131). A more specific strategy depends on the recognition and binding of viral RNA lacking 2′-O methylation of the 5′ RNA cap by IFIT1 (132). This binding ability is enhanced by association with IFIT2 and IFIT3 (133). Despite the fact that CMV replication takes place wholly within the nucleus, export of viral mRNAs does occur (134) and these may be sensed by IFITs. Another possibility is that IFITs may directly bind essential CMV proteins in the cytoplasm, as IFIT1 does to inhibit human papilloma virus (HPV) infection (135, 136). Although the mechanisms of the IFIT-mediated antiviral response to HCMV are still unclear, a significant reduction in titer has been reported when the virus is grown in IFIT1 overexpressing fetal astrocytes (137).

#### IFITMs

IFITM proteins are also implicated in the antiviral response against a wide range of viruses: orthomyxoviruses, flaviviruses, filoviruses, and coronaviruses often by blocking membrane fusion (127, 138–140). However, overexpression of IFITM1, IFITM2 and IFITM3 does not inhibit HCMV infection but rather results in a modest increase in the percentage of infected cells (141, 142). Short hairpin RNA (shRNA) knockdown of IFITM1 alone or in combination with IFITM2 and IFITM3 inhibits HCMV infection as they are required for successful formation of the HCMV virion assembly complex (vAC) and production of infectious progeny virions (142). It is interesting to note that despite this proviral role, IFITM proteins are noticeably downregulated at later stages of infection (48–72 hpi) (142).

Direct induction of IFITMs by HCMV may also contribute to the severe consequences of congenital infection as IFITM expression can inhibit the fusion of cytotrophoblast cells into the multinucleated syncytiotrophoblast, a structure at the interface between maternal and fetal tissue, essential for placental development (143).

#### MxA and MxB

The Mx proteins MxA and MxB are a family of dynamin-like GTPases first reported for their antiviral activity against influenza and are now well characterized in response to other viruses (144, 145). MxA is found in the cytosol and inhibits influenza virus infection through retention of the viral genome (146). On the other hand, MxB localizes to the cytoplasmic face of nuclear pores (147) and is able to inhibit HIV-1 replication by blocking nuclear viral genome accumulation (148, 149). Both MxA and MxB are highly upregulated by HCMV infection (73, 150) and it has recently been discovered that MxB overexpression inhibits replication of HSV-1, HSV-2, Kaposi's sarcoma-associated herpesvirus (KSHV), MCMV, and HCMV (151, 152). HSV-1 and MCMV inhibition manifested in a similar way to that of HIV-1, a block in the delivery of viral genome to the nucleus (151). However, in terms of the regions of protein at play, this mechanism was found to differ substantially with a requirement for GTP binding but not GTP hydrolysis (152, 153). Knockdown of MxB has also been implicated in stalling cell cycle progression (147) and it has been suggested that the HCMV virion protein pUL69 that contributes to the cell cycle arrest (154) does so via an interaction with MxB (155).

## Alternate IFN-Independent Pathways of Innate Response Induction

Direct ISG induction by IRF3 is not the only pathway associated with the IFN-independent response to CMV. In human monocytes, IFN-independent, biphasic activation of STAT1 with differential phosphorylation at early (30 min) compared to late (24 h) time points post-HCMV infection appears to influence motility, migration, differentiation and polarization (156).

Regulation of mitochondrial activity is emerging as another IFN-independent innate response mediator. A number of years ago it was discovered that HCMV DNA could induce ISG expression in an IRF3-dependent, TLR-independent manner that involved TANK-binding kinase 1 (TBK1), IκB kinase epsilon [IKKε; originally called IKK-inducible (IKKi)], and mitochondrial antiviral-signaling protein (MAVS) (157). More recently, peroxisomal MAVS has been implicated in rapid type-I IFN-independent ISG (viperin, Mx2, IFIT3, IFIT2) expression (158). Conversely, mitochondrial MAVS appears to be involved in IFN-dependent ISG production (158). HCMV actively impairs mitochondrial MAVS signaling through the viral mitochondria-localized inhibitor of apoptosis (vMIA) and reduces type I IFN production (159). vMIA has also been found to localize to peroxisomes and induce their fragmentation by interaction with the cytoplasmic chaperone protein Pex19, hijacking the transport machinery of peroxisomal membrane proteins (160). This suggests that disabling IFN-independent ISG transcription induced by peroxisomal MAVS contributes to efficient CMV infection.

The HCMV immediate early gene 1 (IE1) is also capable of inducing expression of ISGs in the absence of IFN production. HCMV IE1 induces expression of IL-6 (161) which usually signals through JAK and STAT3 (162). However, IE1 binds and sequesters STAT3 (163), leaving JAK, already activated by IL-6, free to phosphorylate STAT1. Thus IE1 re-routes IL-6 signaling to activate STAT1 resulting in transcription of ISGs independently of IFN (164).

## Functional Importance of IFN-Independent Innate Responses

Early studies examining IFN-independent induction of an antiviral state showed that treatment of human embryonic lung fibroblasts (HELFs) with UV-HCMV rendered these cells resistant to subsequent viral infection in the absence of detectable IFN production (91). Intriguingly, whilst high multiplicity of infection (MOI) UV-HCMV also induced an antiviral state in the HELFs, this required IFN production (91). Paladino et al. (91) proposed a model by which, when cells are exposed to limited numbers of virus particles (low MOI), induction of an internal antiviral state is sufficient to control infection, however, when many virus particles are present (high MOI), cells secrete IFN to protect neighboring cells too. The ability to induce an antiviral state in the absence of IFN production may be important in cells such as neurons, where inflammation is undesirable. In this respect, neurotropic arboviruses have been shown to induce protective type I IFN-independent, IRF3-dependent responses (165).

Recently, the power of IFN-independent innate responses to CMV has been illustrated by the finding that human macrophages co-cultured with HCMV-infected retinal pigment epithelial cells (RPEs) can limit viral replication and spread in a cell-cell contact dependent manner that could not be blocked by vaccinia-derived type I IFN binding protein B18R, nor by neutralizing antibodies against either IFNγ or TNFα (166). It has also been shown that HCMV virus particles pre-treated with HCMV-specific antibodies that do not replicate, nor express IE antigens, can enter human macrophages and induce an antiviral state that renders these cells less susceptible to subsequent HCMV infection independently of IFN production (167).

IFN-independent ISG induction can also be used to regulate the development of cells key to viral persistence and dissemination. In human monocytes infected with HCMV, ISGs are upregulated independently of IFN (4 hpi) that function to enhance monocyte motility and migration (156). This occurs in a STAT1-dependent manner that also suppresses transcription of anti-inflammatory M2-associated cytokines (IL-10 and CCL18), promoting polarization of macrophages toward a mixed M1/M2 phenotype (156). ISG15 was among the ISGs found to be upregulated in monocytes 4hpi with HCMV (156). ISG15 may contribute both directly and indirectly to the mixed M1/M2 macrophage phenotype, causing monocyte-specific upregulation of IL-10 (168) whilst simultaneously inducing production of M1 macrophage-stimulating cytokine IFNγ by NK and T cells (114).

## Concluding Remarks

When considering the innate response to CMV infection, IFN and the ISG-mediated induction of an antiviral state are important first elements. The intention of this review has been to highlight the substantial body of literature accumulating around IFN-independent innate responses to CMV. IFN-independent induction of ISGs is an important phenomenon and ISGs produced via this pathway appear to play both pro- and anti-viral roles during infection. This complicates direct interrogation of the IFN response during viral infection and necessitates careful consideration of kinetics, as particular ISG may be upregulated directly by the virus, independently of IFN, to play a proviral role early in infection but later on, when IFN-dependent expression dominates, may antagonize infection.

When examining plaque number and size at 7 days post infection, we reported no difference in rates of CMV replication nor spread between cells unable to produce IFN (IRF3 degraded) and those that could not respond to IFN (lacking STAT1) (92), even though the latter cells still allowed viral induction of IFN-independent ISGs (96). Focusing on earlier time points, before loss of IFN production/signaling becomes the overwhelming factor affecting infection efficiency, may reveal more subtle differences conferred by abrogation of either IRF3 or STAT1 signaling.

A deeper understanding of the various functions of IFN-independent ISGs may enable their relative abundance to serve as a predictor of disease progression. For example, high levels of IL-6 have been correlated with CMV reactivation and poor prognosis for transplant patients (169–171); perhaps this is because ISGs produced by IE1 re-routing the IL-6 response to enhance infection. If this were the case, interference with STAT1 homodimer-mediated ISG expression may improve prognosis.

Since CMV infected, polarized macrophages are key mediators of T cell activation and proliferation (172), if IFN-independent ISGylation influences macrophage polarization then levels of ISG15 induced directly by CMV early in infection may provide an indication as to whether or not a robust T cell response will be generated.

It is also important to note that many of these ISGs, including viperin, IFIT2, IFIT3, Mx1 and ISG15 are defined as part of the 28 core mammalian ISGs i.e., produced in all nine mammalian species tested (54). It would therefore be prudent to determine whether their IFN independence is also conserved across species especially since rhesus CMV does not induce IRF3 activation nor the associated ISG expression (173).

Finally, with the IFN-independent nature of these ISGs becoming clear, caution should be exercised when using these ISGs as surrogate readouts for interferon signaling, as it is clear that they are also induced directly by viral infection.

## Author Contributions

CA generated the initial draft of the manuscript. All other authors (BM, AA and BS) contributed to the subsequent writing and review of the manuscript.

### Conflict of Interest

The authors declare that the research was conducted in the absence of any commercial or financial relationships that could be construed as a potential conflict of interest.

## References

[1] Dolan A Cunningham C Hector RD Hassan-Walker AF Lee L Addison C. Genetic content of wild-type human cytomegalovirus. J Gen Virol. (2004) 85(Pt 5):1301–12. 10.1099/vir.0.79888-0 15105547

[2] Stern-Ginossar N Weisburd B Michalski A Le VT Hein MY Huang SX. Decoding human cytomegalovirus. Science. (2012) 338:1088–93. 10.1126/science.1227919 23180859 PMC3817102

[3] Mujtaba G Shaukat S Angez M Alam MM Hasan F Zahoor Zaidi SS. Seroprevalence of Human Cytomegalovirus (HCMV) infection in pregnant women and outcomes of pregnancies with active infection. J Pak Med Assoc. (2016) 66:1009–14. 27524538

[4] Seale H MacIntyre CR Gidding HF Backhouse JL Dwyer DE Gilbert L. National serosurvey of cytomegalovirus in Australia. Clin Vacc Immunol. (2006) 13:1181–4. 10.1128/CVI.00203-06 16957061 PMC1656547

[5] Lachmann R Loenenbach A Waterboer T Brenner N Pawlita M Michel A. Cytomegalovirus (CMV) seroprevalence in the adult population of Germany. PLoS ONE. (2018) 13:e0200267. 10.1371/journal.pone.0200267 30044826 PMC6059406

[6] Cannon MJ Schmid DS Hyde TB Review of cytomegalovirus seroprevalence and demographic characteristics associated with infection. Rev Med Virol. (2010) 20:202–13. 10.1002/rmv.655 20564615

[7] Cannon MJ Griffiths PD Aston V Rawlinson WD. Universal newborn screening for congenital CMV infection: what is the evidence of potential benefit? Rev. Med. Virol. (2014) 24:291–307. 10.1002/rmv.1790 24760655 PMC4494732

[8] Brodin P Jojic V Gao T Bhattacharya S Angel CJ Furman D. Variation in the human immune system is largely driven by non-heritable influences. Cell. (2015) 160:37–47. 10.1016/j.cell.2014.12.020 25594173 PMC4302727

[9] Wallace DL Masters JE De Lara CM Henson SM Worth A Zhang Y. Human cytomegalovirus-specific CD8(+) T-cell expansions contain long-lived cells that retain functional capacity in both young and elderly subjects. Immunology. (2011) 132:27–38. 10.1111/j.1365-2567.2010.03334.x 20738423 PMC3015072

[10] Weekes MP Carmichael AJ Wills MR Mynard K Sissons J. Human CD28(-)CD8(+) T cells contain greatly expanded functional virus-specific memory CTL clones. J Immunol. (1999) 162:7569–77. 10358214

[11] van de Berg PJEJ van Stijn A ten Berge IJM van Lier RAW A fingerprint left by cytomegalovirus infection in the human T cell compartment. J Clin Virol. (2008) 41:213–7. 10.1016/j.jcv.2007.10.016 18061537

[12] Sylwester AW Mitchell BL Edgar JB Taormina C Pelte C Ruchti F. Broadly targeted human cytomegalovirus-specific CD4+ and CD8+ T cells dominate the memory compartments of exposed subjects. J Exp Med. (2005) 202:673–85. 10.1084/jem.20050882 16147978 PMC2212883

[13] Looney RJ Falsey A Campbell D Torres A Kolassa J Brower C. Role of cytomegalovirus in the T cell changes seen in elderly individuals. Clin. Immunol. (1999) 90:213–9. 10.1006/clim.1998.4638 10080833

[14] Amsler L Verweij M DeFilippis VR. The tiers and dimensions of evasion of the type I interferon response by human cytomegalovirus. J Mol Biol. (2013) 425:4857–71. 10.1016/j.jmb.2013.08.023 24013068 PMC3864659

[15] Biolatti M Gugliesi F Dell'Oste V Landolfo S. Modulation of the innate immune response by human cytomegalovirus. Infect Genet Evol. (2018) 64:105–14. 10.1016/j.meegid.2018.06.025 29935337

[16] Goodwin CM Ciesla JH Munger J. Who's driving? human cytomegalovirus, interferon, and NFκB signaling. Viruses. (2018) 10:E447. 10.3390/v10090447 30134546 PMC6163874

[17] Galitska G Biolatti M Griffante G Gugliesi F Pasquero S Dell'Oste V Catch me if you can: the arms race between human cytomegalovirus and the innate immune system. Fut Virol. (2019) 14:247–63. 10.2217/fvl-2018-0189

[18] Murray MJ Peters NE Reeves MB. Navigating the host cell response during entry into sites of latent cytomegalovirus infection. Pathogens. (2018) 7:30. 10.3390/pathogens7010030 29547547 PMC5874756

[19] Rossini G Cerboni C Santoni A Landini MP Landolfo S Gatti D Interplay between human cytomegalovirus and intrinsic/innate host responses: a complex bidirectional relationship 2012. Mediators Inflamm. (2012) 2012:607276 10.1155/2012/607276 22701276 PMC3371353

[20] Krmpotic A Bubic I Polic B Lucin P Jonjic S. Pathogenesis of murine cytomegalovirus infection. Microb Infect. (2003) 5:1263–77. 10.1016/j.micinf.2003.09.007 14623023

[21] Egli A Levin A Santer DM Joyce M O'Shea D Thomas BS. Immunomodulatory function of interleukin 28B during primary infection with cytomegalovirus. J Infect Dis. (2014) 210:717–27. 10.1093/infdis/jiu144 24620020

[22] Brand S Beigel F Olszak T Zitzmann K Eichhorst ST Otte JM. IL-28A and IL-29 mediate antiproliferative and antiviral signals in intestinal epithelial cells and murine CMV infection increases colonic IL-28A expression. Am J Physiol Gastrointest Liver Physiol. (2005) 289:G960–8. 10.1152/ajpgi.00126.2005 16051921

[23] Annibali O Piccioni L Tomarchio V Circhetta E Sarlo C Franceschini L. Impact of IFN lambda 3/4 single nucleotide polymorphisms on the cytomegalovirus reactivation in autologous stem cell transplant patients. PLoS ONE. (2018) 13:e0200221. 10.1371/journal.pone.0200221 30036376 PMC6056038

[24] Manuel O Wójtowicz A Bibert S Mueller NJ van Delden C Hirsch HH. Influence of IFNL3/4 polymorphisms on the incidence of cytomegalovirus infection after solid-organ transplantation. J Infect Dis. (2014) 211:906–14. 10.1093/infdis/jiu557 25301956

[25] Bibert S Wojtowicz A Taffe P Manuel O Bernasconi E Furrer H. The IFNL3/4 DeltaG variant increases susceptibility to cytomegalovirus retinitis among HIV-infected patients. Aids. (2014) 28:1885–9. 10.1097/QAD.0000000000000379 25259701

[26] Gimeno Brias S Marsden M Forbester J Clement M Brandt C Harcourt K Interferon lambda is required for interferon gamma-expressing NK cell responses but does not afford antiviral protection during acute and persistent murine cytomegalovirus infection. PLoS ONE. (2018) 13:e0197596 10.1371/journal.pone.0197596 29768502 PMC5955543

[27] Ding S Khoury-Hanold W Iwasaki A Robek MD. Epigenetic Reprogramming of the type III interferon response potentiates antiviral activity and suppresses tumor growth. PLoS Biol. (2014) 12:e1001758. 10.1371/journal.pbio.1001758 24409098 PMC3883642

[28] Boehme KW Singh J Perry ST Compton T. Human cytomegalovirus elicits a coordinated cellular antiviral response via envelope glycoprotein. J Virol B. (2004) 78:1202–11. 10.1128/JVI.78.3.1202-1211.2004 14722275 PMC321386

[29] Boehme KW Guerrero M Compton T. Human cytomegalovirus envelope glycoproteins B and H are necessary for TLR2 activation in permissive cells. J Immunol. (2006) 177:7094–102. 10.4049/jimmunol.177.10.7094 17082626

[30] Compton T Kurt-Jones EA Boehme KW Belko J Latz E Golenbock DT. Human cytomegalovirus activates inflammatory cytokine responses via CD14 and Toll-like receptor 2. J Virol. (2003) 77:4588–96. 10.1128/JVI.77.8.4588-4596.2003 12663765 PMC152130

[31] Szomolanyi-Tsuda E Liang X Welsh RM Kurt-Jones EA Finberg RW. Role for TLR2 in NK cell-mediated control of murine cytomegalovirus *in vivo*. J Virol. (2006) 80:4286–91. 10.1128/JVI.80.9.4286-4291.2006 16611887 PMC1472014

[32] Paijo J Döring M Spanier J Grabski E Nooruzzaman M Schmidt T. cGAS senses human cytomegalovirus and induces type I interferon responses in human monocyte-derived cells. PLoS Pathog. (2016) 12:e1005546. 10.1371/journal.ppat.1005546 27058035 PMC4825940

[33] Varani S Cederarv M Feld S Tammik C Frascaroli G Landini MP. Human cytomegalovirus differentially controls B cell and T cell responses through effects on plasmacytoid dendritic cells. J Immunol. (2007) 179:7767–76. 10.4049/jimmunol.179.11.7767 18025223

[34] Krug A French AR Barchet W. J. Fischer AA Dzionek A Pingel JT. TLR9-dependent recognition of MCMV by IPC and DC generates coordinated cytokine responses that activate antiviral NK cell function. Immunity. (2004) 21:107–19. 10.1016/j.immuni.2004.06.007 15345224

[35] Gariano GR. V. Dell'Oste Bronzini M Gatti D Luganini A De Andrea M. The intracellular DNA sensor IFI16 gene acts as restriction factor for human cytomegalovirus replication. PLoS Pathog. (2012) 8:e1002498. 10.1371/journal.ppat.1002498 22291595 PMC3266931

[36] Rolle S De Andrea M Gioia D Lembo D Hertel L Landolfo S. The interferon-inducible 204 gene is transcriptionally activated by mouse cytomegalovirus and is required for its replication. Virology. (2001) 286:249–55. 10.1006/viro.2001.1021 11485393

[37] DeFilippis VR Alvarado D Sali T Rothenburg S Früh K. Human cytomegalovirus induces the interferon response via the DNA sensor ZBP1. J Virol. (2010) 84:585–98. 10.1128/JVI.01748-09 19846511 PMC2798427

[38] DeFilippis VR Sali T Alvarado D White L Bresnahan W Früh KJ Activation of the interferon response by human cytomegalovirus occurs via cytoplasmic double-stranded DNA but not glycoprotein B. J Virol. (2010) 84:8913–25. 10.1128/JVI.00169-10 20573816 PMC2919031

[39] Upton JW William KJ Edward MS. DAI/ZBP1/DLM-1 complexes with RIP3 to mediate virus-induced programmed necrosis that is targeted by murine cytomegalovirus vIRA. Cell Host Microbe. (2012) 11:290–7. 10.1016/j.chom.2012.01.016 22423968 PMC3531981

[40] Lio CWJ McDonald B Takahashi M Dhanwani R Sharma N Huang J. cGAS-STING signaling regulates initial innate control of cytomegalovirus infection. J Virol. (2016) 90:7789–97. 10.1128/JVI.01040-16 27334590 PMC4988162

[41] Au WC Moore PA Lowther W Juang YT Pitha PM. Identification of a member of the interferon regulatory factor family that binds to the interferon-stimulated response element and activates expression of interferon-induced genes. Proc Natl Acad Sci USA. (1995) 92:11657–61. 10.1073/pnas.92.25.11657 8524823 PMC40461

[42] DeFilippis VR Robinson B Keck TM Hansen SG Nelson JA Früh KJ. Interferon regulatory factor 3 is necessary for induction of antiviral genes during human cytomegalovirus infection. J Virol. (2006) 80:1032–7. 10.1128/JVI.80.2.1032-1037.2006 16379004 PMC1346858

[43] Takaoka A Wang Z Choi MK Yanai H Negishi H Ban T. DAI (DLM-1/ZBP1) is a cytosolic DNA sensor and an activator of innate immune response. Nature. (2007) 448:501–5. 10.1038/nature06013 17618271

[44] Zhang X Shi H Wu J Zhang X Sun L Chen C Cyclic GMP-AMP containing mixed phosphodiester linkages is an endogenous high-affinity ligand for STIN. Mol Cell. (2013) 51:226–35. 10.1016/j.molcel.2013.05.022 23747010 PMC3808999

[45] Marie I Durbin JE Levy DE. Differential viral induction of distinct interferon-alpha genes by positive feedback through interferon regulatory factor-7. Embo J. (1998) 17:6660–9. 10.1093/emboj/17.22.6660 9822609 PMC1171011

[46] Vieira Braga FA Hertoghs KM van Lier RA van Gisbergen KP. Molecular characterization of HCMV-specific immune responses: Parallels between CD8(+) T cells, CD4(+) T cells, and NK cells. Eur J Immunol. (2015) 45:2433–45. 10.1002/eji.201545495 26228786

[47] Gamadia LE Remmerswaal EBM Weel JF Bemelman F van Lier RAW Ten Berge IJM. Primary immune responses to human CMV: a critical role for IFN-γ-producing CD4+ T cells in protection against CMV disease. Blood. (2003) 101:2686–92. 10.1182/blood-2002-08-2502 12411292

[48] Quinn M Turula H Tandon M Deslouches B Moghbeli T Snyder CM. Memory T cells specific for murine cytomegalovirus re-emerge after multiple challenges and recapitulate immunity in various adoptive transfer scenarios. J Immunol. (2015) 194:1726–36. 10.4049/jimmunol.1402757 25595792 PMC4684174

[49] Foley B Cooley S Verneris MR Pitt M Curtsinger J Luo X. Cytomegalovirus reactivation after allogeneic transplantation promotes a lasting increase in educated NKG2C+ natural killer cells with potent function. Blood. (2012) 119:2665–74. 10.1182/blood-2011-10-386995 22180440 PMC3311280

[50] Luetke-Eversloh M Hammer Q Durek P Nordström K Gasparoni G Pink M. Human cytomegalovirus drives epigenetic imprinting of the IFNG locus in NKG2Chi natural killer cells. PLoS Pathog. (2014) 10:e1004441. 10.1371/journal.ppat.1004441 25329659 PMC4199780

[51] Ank N West H Bartholdy C Eriksson K Thomsen AR Paludan SR. Lambda interferon (IFN-λ), a type III IFN, is induced by viruses and IFNs and displays potent antiviral activity against select virus infections *in vivo*. J Virol. (2006) 80:4501–9. 10.1128/JVI.80.9.4501-4509.2006 16611910 PMC1472004

[52] Schoggins JW Interferon-stimulated genes: what do they all do? Annu Rev Virol. (2019) 6:567–84. 10.1146/annurev-virology-092818-015756 31283436

[53] Rusinova I Forster S Yu S Kannan A Masse M Cumming H. INTERFEROME v2.0: an updated database of annotated interferon-regulated genes. Nucl Acids Res. (2012). 41:D1040–6. 10.1093/nar/gks1215 23203888 PMC3531205

[54] Shaw AE Hughes J Gu Q Behdenna A Singer JB Dennis T. Fundamental properties of the mammalian innate immune system revealed by multispecies comparison of type I interferon responses. PLoS Biol. (2017) 15:e2004086. 10.1371/journal.pbio.2004086 29253856 PMC5747502

[55] Zhou Z Hamming OJ Ank N Paludan SR Nielsen AL Hartmann R. Type III interferon (IFN) induces a type I IFN-like response in a restricted subset of cells through signaling pathways involving both the Jak-STAT pathway and the mitogen-activated protein kinases. J Virol. (2007) 81:7749. 10.1128/JVI.02438-06 17507495 PMC1933366

[56] Sommereyns C Paul S Staeheli P Michiels T. IFN-lambda (IFN-lambda) is expressed in a tissue-dependent fashion and primarily acts on epithelial cells *in vivo*. PLoS Pathog. (2008) 4:e1000017. 10.1371/journal.ppat.1000017 18369468 PMC2265414

[57] Darnell JE Jr. Kerr IM Stark GR. Jak-STAT pathways and transcriptional activation in response to IFNs and other extracellular signaling proteins. Science. (1994) 264:1415–21. 10.1126/science.8197455 8197455

[58] Schindler C Shuai K Prezioso VR Darnell JE Jr. Interferon-dependent tyrosine phosphorylation of a latent cytoplasmic transcription factor. Science. (1992) 257:809–13. 10.1126/science.1496401 1496401

[59] Levy DE Kessler DS Pine R Darnell JE Jr Cytoplasmic activation of ISGF3, the positive regulator of interferon-alpha-stimulated transcription, reconstituted *in vitro*. Genes Dev. (1989) 3:1362–71. 10.1101/gad.3.9.1362 2606351

[60] Fu XY. A transcription factor with SH2 and SH3 domains is directly activated by an interferon alpha-induced cytoplasmic protein tyrosine kinase(s). Cell. (1992) 70:323–35. 10.1016/0092-8674(92)90106-M 1638633

[61] Levy DE Kessler DS Pine R Reich N Darnell JE Jr. Interferon-induced nuclear factors that bind a shared promoter element correlate with positive and negative transcriptional control. Genes Dev. (1988) 2:383–93. 10.1101/gad.2.4.383 3371658

[62] Kotenko SV Gallagher G Baurin VV Lewis-Antes A Shen M Shah NK. IFN-λs mediate antiviral protection through a distinct class II cytokine receptor complex. Nat Immunol. (2003) 4:69–77. 10.1038/ni875 12483210

[63] Poat B Hazari S Chandra PK Gunduz F Alvarez X Balart LA. Intracellular expression of IRF9 Stat fusion protein overcomes the defective Jak-Stat signaling and inhibits HCV RNA replication. Virol J. (2010) 7:265. 10.1186/1743-422X-7-265 20939906 PMC2964675

[64] Kraus TA Lau JF Parisien JP Horvath CM. A hybrid IRF9-STAT2 protein recapitulates interferon-stimulated gene expression and antiviral response. J Biol Chem. (2003) 278:13033–38. 10.1074/jbc.M212972200 12574168

[65] Fink K Grandvaux N. STAT2 and IRF9. JAK-STAT. (2013) 2:e27521. 10.4161/jkst.27521 24498542 PMC3906322

[66] Shuai K Schindler C Prezioso V Darnell J. Activation of transcription by IFN-gamma: tyrosine phosphorylation of a 91-kD DNA binding protein. Science. (1992) 258:1808–12. 10.1126/science.1281555 1281555

[67] Decker T Lew DJ Mirkovitch J Darnell JE Jr. Cytoplasmic activation of GAF, an IFN-gamma-regulated DNA-binding factor. Embo J. (1991) 10:927–32. 10.1002/j.1460-2075.1991.tb08026.x 1901265 PMC452736

[68] Decker T Kovarik P Meinke A. GAS elements: a few nucleotides with a major impact on cytokine-induced gene expression. J Interferon Cytokine Res. (1997) 17:121–34. 10.1089/jir.1997.17.121 9085936

[69] Decker T Lew DJ Cheng YS Levy DE Darnell JE Jr. Interactions of alpha- and gamma-interferon in the transcriptional regulation of the gene encoding a guanylate-binding protein. Embo J. (1989) 8:2009–14. 10.1002/j.1460-2075.1989.tb03608.x 2507314 PMC401078

[70] Pearse RN Feinman R Ravetch JV. Characterization of the promoter of the human gene encoding the high-affinity IgG receptor: transcriptional induction by gamma-interferon is mediated through common DNA response elements. Proc Natl Acad Sci USA. (1991) 88:11305–9. 10.1073/pnas.88.24.11305 1837149 PMC53123

[71] Zhu H Cong JP Mamtora G Gingeras T Shenk T. Cellular gene expression altered by human cytomegalovirus: global monitoring with oligonucleotide arrays. Proc Natl Acad Sci USA. (1998) 95:14470–5. 10.1073/pnas.95.24.14470 9826724 PMC24397

[72] Zhu H Cong JP Shenk T. Use of differential display analysis to assess the effect of human cytomegalovirus infection on the accumulation of cellular RNAs: Induction of interferon-responsive RNAs. Proc Natl Acad Sci USA. (1997) 94:13985–90. 10.1073/pnas.94.25.13985 9391139 PMC28419

[73] Browne EP Wing B Coleman D Shenk T. Altered cellular mRNA levels in human cytomegalovirus-infected fibroblasts: viral block to the accumulation of antiviral mRNAs. J Virol. (2001) 75:12319–30. 10.1128/JVI.75.24.12319-12330.2001 11711622 PMC116128

[74] Grandvaux N Servant MJ tenOever B Sen GC Balachandran S Barber GN. Transcriptional profiling of interferon regulatory factor 3 target genes: direct involvement in the regulation of interferon-stimulated genes. J Virol. (2002) 76:5532–9. 10.1128/JVI.76.11.5532-5539.2002 11991981 PMC137057

[75] Wathelet MG Berr PM Huez GA. Regulation of gene expression by cytokines and virus in human cells lacking the type-I interferon locus. Eur J Biochem. (1992) 206:901–10. 10.1111/j.1432-1033.1992.tb16999.x 1318841

[76] Guo J Peters KL Sen GC Induction of the human protein P56 by interferon, double-stranded RNA, or virus infection. Virology. (2000) 267:209–19. 10.1006/viro.1999.0135 10662616

[77] Mossman KL Macgregor PF Rozmus JJ Goryachev AB Edwards AM Smiley JR. Herpes simplex virus triggers and then disarms a host antiviral response. J Virol. (2001) 75:750–8. 10.1128/JVI.75.2.750-758.2001 11134288 PMC113971

[78] Deschamps T Kalamvoki M. Impaired STING pathway in human osteosarcoma U2OS cells contributes to the growth of ICP0-null mutant herpes simplex virus. J Virol. (2017) 91:e00006–17. 10.1128/JVI.00006-17 28179534 PMC5391473

[79] Wu J Sun L Chen X Du F Shi H Chen C. Cyclic GMP-AMP is an endogenous second messenger in innate immune signaling by cytosolic DNScience A. Science. (2013) 339:826–30. 10.1126/science.1229963 23258412 PMC3855410

[80] Sun L Wu J Du F Chen X Chen ZJ. Cyclic GMP-AMP synthase is a cytosolic DNA sensor that activates the type I interferon pathway. Science. (2013) 339:786–91. 10.1126/science.1232458 23258413 PMC3863629

[81] Ablasser A Schmid-Burgk JL Hemmerling I Horvath GL Schmidt T Latz E Cell intrinsic immunity spreads to bystander cells via the intercellular transfer of cGAMNature P. Nature. (2013) 503:530–4. 10.1038/nature12640 24077100 PMC4142317

[82] Liu S Cai X Wu J Cong Q Chen X Li T. Phosphorylation of innate immune adaptor proteins MAVS, STING, and TRIF induces IRF3 activation. Science. (2015) 347:aaa2630. 10.1126/science.aaa2630 25636800

[83] Collins SE Noyce RS Mossman KL Innate cellular response to virus particle entry requires IRF3 but not virus replication. J Virol. (2004) 78:1706–17. 10.1128/JVI.78.4.1706-1717.2004 14747536 PMC369475

[84] Guo J Hui DJ Merrick WC Sen GC. A new pathway of translational regulation mediated by eukaryotic initiation factor 3. EMBO J. (2000) 19:6891–9. 10.1093/emboj/19.24.6891 11118224 PMC305884

[85] Navarro L Mowen K Rodems S Weaver B Reich N Spector D. Cytomegalovirus activates interferon immediate-early response gene expression and an interferon regulatory factor 3-containing interferon-stimulated response element-binding complex. Mol Cell Biol. (1998) 18:3796–02. 10.1128/MCB.18.7.3796 9632763 PMC108963

[86] Preston CM Harman AN Nicholl MJ Activation of interferon response factor-3 in human cells infected with herpes simplex virus type 1 or human cytomegalovirus. J Virol. (2001) 75:8909–16. 10.1128/JVI.75.19.8909-8916.2001 11533154 PMC114459

[87] Noyce RS Collins SE Mossman KL. Identification of a novel pathway essential for the immediate-early, interferon-independent antiviral response to enveloped virions. J Virol. (2006) 80:226–35. 10.1128/JVI.80.1.226-235.2006 16352547 PMC1317555

[88] Chin KC Cresswell P. Viperin (cig5), an IFN-inducible antiviral protein directly induced by human cytomegalovirus. Proc Natl Acad Sci USA. (2001) 98:15125–30. 10.1073/pnas.011593298 11752458 PMC64994

[89] Stirnweiss A Ksienzyk A Klages K Rand U Grashoff M Hauser H. IFN regulatory factor-1 bypasses IFN-mediated antiviral effects through viperin gene induction. J Immunol. (2010) 184:5179–85. 10.4049/jimmunol.0902264 20308629

[90] Jin J Hu H Li HS Yu J Xiao Y Brittain GC. Noncanonical NF-κB pathway controls the production of type I interferons in antiviral innate immunity. Immunity. (2014) 40:342–54. 10.1016/j.immuni.2014.02.006 24656046 PMC3983709

[91] Paladino P Cummings DT Noyce RS Mossman KL. The IFN-independent response to virus particle entry provides a first line of antiviral defense that is independent of TLRs and retinoic acid-inducible gene I. J Immunol. (2006) 177:8008–16. 10.4049/jimmunol.177.11.8008 17114474

[92] McSharry BP Forbes SK Avdic S Randall RE Wilkinson GW Abendroth A. Abrogation of the interferon response promotes more efficient human cytomegalovirus replication. J Virol. (2015) 89:1479–83. 10.1128/JVI.02988-14 25392213 PMC4300662

[93] McSharry BP Forbes SK Cao JZ Avdic S Machala EA Gottlieb DJ. Human cytomegalovirus upregulates expression of the lectin galectin 9 via induction of beta interferon. J Virol. (2014) 88:10990–4. 10.1128/JVI.01259-14 25008927 PMC4178876

[94] Hilton L Moganeradj K Zhang G Chen YH Randall RE McCauley JW. The NPro product of bovine viral diarrhea virus inhibits DNA binding by interferon regulatory factor 3 and targets it for proteasomal degradation. J Virol. (2006) 80:11723–32. 10.1128/JVI.01145-06 16971436 PMC1642611

[95] Andrejeva J Young DF Goodbourn S Randall RE. Degradation of STAT1 and STAT2 by the V proteins of simian virus 5 and human parainfluenza virus type 2, respectively: consequences for virus replication in the presence of alpha/beta and gamma interferons. J Virol. (2002) 76:2159–67. 10.1128/jvi.76.5.2159-2167.2002 11836393 PMC153821

[96] Ashley CL Abendroth A McSharry BP Slobedman B. Interferon-independent upregulation of interferon-stimulated genes during human cytomegalovirus infection is dependent on IRF3 expression. Viruses. (2019) 11:246. 10.3390/v11030246 30871003 PMC6466086

[97] Van der Hoek KH Eyre NS Shue B Khantisitthiporn O Glab-Ampi K Carr JM. Viperin is an important host restriction factor in control of Zika virus infection. Sci Rep. (2017) 7:4475. 10.1038/s41598-017-04138-1 28667332 PMC5493656

[98] Wang X Hinson ER Cresswell P. The interferon-inducible protein viperin inhibits influenza virus release by perturbing lipid rafts. Cell Host Microbe. (2007) 2:96–105. 10.1016/j.chom.2007.06.009 18005724

[99] Rivieccio MA Suh HS Zhao Y Zhao ML Chin KC Lee SC. TLR3 ligation activates an antiviral response in human fetal astrocytes: a role for viperin/cig5. J Immunol. (2006) 177:4735–41. 10.4049/jimmunol.177.7.4735 16982913

[100] Zhang Y Burke CW Ryman KD Klimstra WB. Identification and characterization of interferon-induced proteins that inhibit alphavirus replication. J Virol. (2007) 81:11246–55. 10.1128/JVI.01282-07 17686841 PMC2045553

[101] Helbig KJ Lau DT Semendric L Harley HA Beard MR. Analysis of ISG expression in chronic hepatitis C identifies viperin as a potential antiviral effector. Hepatology. (2005) 42:702–10. 10.1002/hep.20844 16108059

[102] Helbig KJ Beard MR. The role of viperin in the innate antiviral response. J Mol Biol. (2014) 426:1210–9. 10.1016/j.jmb.2013.10.019 24157441

[103] Seo JY Cresswell P. Viperin regulates cellular lipid metabolism during human cytomegalovirus infection. PLoS Pathog. (2013) 9:e1003497. 10.1371/journal.ppat.1003497 23935494 PMC3731232

[104] Halwachs-Baumann G Wilders-Truschnig M Desoye G Hahn T Kiesel L Klingel K. Human trophoblast cells are permissive to the complete replicative cycle of human cytomegalovirus. J Virol. (1998) 72:7598–602. 9696860 10.1128/jvi.72.9.7598-7602.1998 PMC110014

[105] Tabata T Petitt M Zydek M Fang-Hoover J Larocque N Tsuge M. Human cytomegalovirus infection interferes with the maintenance and differentiation of trophoblast progenitor cells of the human placenta. J Virol. (2015) 89:5134–47. 10.1128/JVI.03674-14 25741001 PMC4403461

[106] Hemmings DG Kilani R Nykiforuk C Preiksaitis J Guilbert LJ. Permissive cytomegalovirus infection of primary villous term and first trimester trophoblasts. J Virol. (1998) 72:4970–9. 9573266 10.1128/jvi.72.6.4970-4979.1998 PMC110059

[107] Wang B Fang Y Wu Y Koga K Osuga Y Lv S. Viperin is induced following toll-like receptor 3 (TLR3) ligation and has a virus-responsive function in human trophoblast cells. Placenta. (2015) 36:667–73. 10.1016/j.placenta.2015.03.002 25814471

[108] Saitoh T Satoh T Yamamoto N Uematsu S Takeuchi O Kawai T. Antiviral protein viperin promotes toll-like receptor 7- and toll-like receptor 9-mediated type i interferon production in plasmacytoid dendritic cells. Immunity. (2011) 34:352–63. 10.1016/j.immuni.2011.03.010 21435586

[109] Gizzi AS Grove TL Arnold JJ Jose J Jangra RK Garforth SJ. A naturally occurring antiviral ribonucleotide encoded by the human genome. Nature. (2018) 558:610–4. 10.1038/s41586-018-0238-4 29925952 PMC6026066

[110] Kambara H Niazi F Kostadinova L Moonka DK Siegel CT Post AB. Negative regulation of the interferon response by an interferon-induced long non-coding RNA. Nucl Acids Res. (2014) 42:10668–80. 10.1093/nar/gku713 25122750 PMC4176326

[111] Bai L Dong J Liu Z Rao Y Feng P Lan K. Viperin catalyzes methionine oxidation to promote protein expression and function of helicases. Sci Adv. (2019) 5:eaax1031. 10.1126/sciadv.aax1031 31489375 PMC6713503

[112] Blomstrom DC Fahey D Kutny R Korant BD Knight E Jr. Molecular characterization of the interferon-induced 15-kDa protein. Molecular cloning and nucleotide and amino acid sequence. J Biol Chem. (1986) 261:8811–6. 3087979

[113] Haas AL Ahrens P Bright PM Ankel H. Interferon induces a 15-kilodalton protein exhibiting marked homology to ubiquitin. J Biol Chem. (1987) 262:11315–23. 2440890

[114] Bogunovic D Byun M Durfee LA Abhyankar A Sanal O Mansouri D. Mycobacterial disease and impaired IFN-gamma immunity in humans with inherited ISG15 deficiency. Science. (2012) 337:1684–8. 10.1126/science.1224026 22859821 PMC3507439

[115] Bianco C Mohr I. Restriction of human cytomegalovirus replication by ISG15, a host effector regulated by cGAS-STING double-stranded-DNA sensing. J Virol. (2017) 91:e02483–16. 10.1128/JVI.02483-16 28202760 PMC5391456

[116] Kim YJ Kim ET Kim YE Lee MK Kwon KM Kim KI. Consecutive inhibition of ISG15 expression and ISGylation by cytomegalovirus regulators. PLoS Pathog. (2016) 12:e1005850. 10.1371/journal.ppat.1005850 27564865 PMC5001722

[117] Lee MK Kim YJ Kim YE Han TH Milbradt J Marschall M. Transmembrane protein pUL50 of human cytomegalovirus inhibits ISGylation by downregulating UBE1L. J Virol. (2018) 92:e00462–18. 10.1128/JVI.00462-18 29743376 PMC6052311

[118] Zimmermann C Büscher N Krauter S Krämer N Wolfrum U Sehn E. The abundant tegument protein pUL25 of human cytomegalovirus prevents proteasomal degradation of pUL26 and supports its suppression of ISGylation. J Virol. (2018) 92:e01180–18. 10.1128/JVI.01180-18 30282718 PMC6258951

[119] Schoggins JW. Interferon-stimulated genes: roles in viral pathogenesis. Curr Opin Virol. (2014) 6:40–6. 10.1016/j.coviro.2014.03.006 24713352 PMC4077717

[120] Speer SD Li Z Buta S Payelle-Brogard B Qian L Vigant F ISG15 deficiency and increased viral resistance in humans but not mice. Nat Commun. (2016) 7:11496 10.1038/ncomms11496 27193971 PMC4873964

[121] Malakhova OA Yan M Malakhov MP Yuan Y Ritchie KJ Kim KI. Protein ISGylation modulates the JAK-STAT signaling pathway. Genes Dev. (2003) 17:455–60. 10.1101/gad.1056303 12600939 PMC195994

[122] Chan G Bivins-Smith ER Smith MS Smith PM Yurochko AD. Transcriptome analysis reveals human cytomegalovirus reprograms monocyte differentiation toward an M1 macrophage. J Immunol. (2008) 181:698–711. 10.4049/jimmunol.181.1.698 18566437 PMC2614917

[123] Smith MS Bentz GL Alexander JS Yurochko AD. Human cytomegalovirus induces monocyte differentiation and migration as a strategy for dissemination and persistence. J Virol. (2004) 78:4444–53. 10.1128/JVI.78.9.4444-4453.2004 15078925 PMC387677

[124] Chan G Nogalski MT Stevenson EV Yurochko AD. Human cytomegalovirus induction of a unique signalsome during viral entry into monocytes mediates distinct functional changes: a strategy for viral dissemination. J Leukocyte Biol. (2012) 92:743–52. 10.1189/jlb.0112040 22715139 PMC3441319

[125] Albert M Bécares M Falqui M Fernández-Lozano C Guerra S. ISG15, a small molecule with huge implications: regulation of mitochondrial homeostasis. Viruses. (2018) 10:629. 10.3390/v10110629 30428561 PMC6265978

[126] Baldanta S Fernández-Escobar M Acín-Perez R Albert M Camafeita E Jorge I. ISG15 governs mitochondrial function in macrophages following vaccinia virus infection. PLoS Pathog. (2017) 13:e1006651. 10.1371/journal.ppat.1006651 29077752 PMC5659798

[127] Diamond MS Farzan M. The broad-spectrum antiviral functions of IFIT and IFITM proteins. Nat Rev Immunol. (2012) 13:46. 10.1038/nri3344 23237964 PMC3773942

[128] Vladimer GI Górna MW Superti-Furga G. IFITs: emerging roles as key anti-viral proteins. Front. Immunol. (2014) 5:94. 10.3389/fimmu.2014.00094 24653722 PMC3948006

[129] Fensterl V Sen GC. Interferon-induced Ifit proteins: their role in viral pathogenesis. J Virol. (2015) 89:2462–8. 10.1128/JVI.02744-14 25428874 PMC4325746

[130] Mears HV Sweeney TR. Better together: the role of IFIT protein-protein interactions in the antiviral response. J Gen Virol. (2018) 99:1463–77. 10.1099/jgv.0.001149 30234477

[131] Wang C Pflugheber J Sumpter R Sodora DL Hui D Sen GC. Alpha interferon induces distinct translational control programs to suppress hepatitis C virus RNA replication. J Virol. (2003) 77:3898–912. 10.1128/JVI.77.7.3898-3912.2003 12634350 PMC150642

[132] Daffis S Szretter KJ Schriewer J Li J Youn S Errett J. 2'-O methylation of the viral mRNA cap evades host restriction by IFIT family members. Nature. (2010) 468:452–6. 10.1038/nature09489 21085181 PMC3058805

[133] Pichlmair A Lassnig C Eberle CA Gorna MW Baumann CL Burkard TR. IFIT1 is an antiviral protein that recognizes 5'-triphosphate RNA. Nat Immunol. (2011) 12:624–30. 10.1038/ni.2048 21642987

[134] Lischka P Toth Z Thomas M Mueller R Stamminger T. The UL69 transactivator protein of human cytomegalovirus interacts with DEXD/H-Box RNA helicase UAP56 to promote cytoplasmic accumulation of unspliced RNA. Mol Cell Biol. (2006) 26:1631–43. 10.1128/MCB.26.5.1631-1643.2006 16478985 PMC1430265

[135] Saikia P Fensterl V Sen GC. The inhibitory action of P56 on select functions of E1 mediates interferon's effect on human papillomavirus DNA replication. J Virol. (2010) 84:13036–9. 10.1128/JVI.01194-10 20926571 PMC3004335

[136] Terenzi F Saikia P Sen GC. Interferon-inducible protein, P56, inhibits HPV DNA replication by binding to the viral protein E1. Embo J. (2008) 27:3311–21. 10.1038/emboj.2008.241 19008854 PMC2609736

[137] Zhang L Wang B Li L Qian DM Yu H Xue ML. Antiviral effects of IFIT1 in human cytomegalovirus-infected fetal astrocytes. J Med Virol. (2017) 89:672–84. 10.1002/jmv.24674 27589693 PMC7166973

[138] Perreira JM Chin CR Feeley EM Brass AL. IFITMs restrict the replication of multiple pathogenic viruses. J Mol Biol. (2013) 425:4937–55. 10.1016/j.jmb.2013.09.024 24076421 PMC4121887

[139] Smith S Weston S Kellam P Marsh M. IFITM proteins-cellular inhibitors of viral entry. Curr Opin Virol. (2014) 4:71–7. 10.1016/j.coviro.2013.11.004 24480526 PMC7185728

[140] Liao Y Goraya MU Yuan X Zhang B Chiu SH Chen JL. Functional involvement of interferon-inducible transmembrane proteins in antiviral immunity. Front Microbiol. (2019) 10:1097. 10.3389/fmicb.2019.01097 31156602 PMC6532022

[141] Warren CJ Griffin LM Little AS Huang IC Farzan M Pyeon D The antiviral restriction factors IFITM1, 2 and 3 do not inhibit infection of human papillomavirus, cytomegalovirus and adenovirus. PLoS ONE. (2014) 9:e96579 10.1371/journal.pone.0096579 24827144 PMC4020762

[142] Xie M Xuan B Shan J Pan D Sun Y Shan Z. Human cytomegalovirus exploits interferon-induced transmembrane proteins to facilitate morphogenesis of the virion assembly compartment. J Virol. (2015) 89:3049–61. 10.1128/JVI.03416-14 25552713 PMC4337551

[143] Buchrieser J Degrelle SA Couderc T Nevers Q Disson O Manet C. IFITM proteins inhibit placental syncytiotrophoblast formation and promote fetal demise. Science. (2019) 365:176–80. 31296770 10.1126/science.aaw7733

[144] Verhelst J Hulpiau P Saelens X. Mx proteins: antiviral gatekeepers that restrain the uninvited. Microbiol Mol Biol Rev. (2013) 77:551–66. 10.1128/MMBR.00024-13 24296571 PMC3973384

[145] Haller O Stertz S Kochs G. The Mx GTPase family of interferon-induced antiviral proteins. Microb Infect. (2007) 9:1636–43. 10.1016/j.micinf.2007.09.010 18062906

[146] Xiao H Killip MJ Staeheli P Randall RE Jackson D. The human interferon-induced MxA protein inhibits early stages of influenza A virus infection by retaining the incoming viral genome in the cytoplasm. J Virol. (2013) 87:13053–8. 10.1128/JVI.02220-13 24049170 PMC3838145

[147] King MC Raposo G Lemmon MA. Inhibition of nuclear import and cell-cycle progression by mutated forms of the dynamin-like GTPase MxB. Proc Natl Acad Sci USA. (2004) 101:8957–62. 10.1073/pnas.0403167101 15184662 PMC428454

[148] Kane M Yadav SS Bitzegeio J Kutluay SB Zang T Wilson SJ. MX2 is an interferon-induced inhibitor of HIV-1 infection. Nature. (2013) 502:563–6. 10.1038/nature12653 24121441 PMC3912734

[149] Goujon C Moncorge O Bauby H Doyle T Ward CC Schaller T. Human MX2 is an interferon-induced post-entry inhibitor of HIV-1 infection. Nature. (2013) 502:559–62. 10.1038/nature12542 24048477 PMC3808269

[150] Pautasso S Galitska G Dell'Oste V Biolatti M Cagliani R Forni D. Strategy of human cytomegalovirus to escape interferon beta-induced APOBEC3G editing activity. J Virol. (2018) 92:e01224–18. 10.1128/JVI.01224-18 30045985 PMC6146821

[151] Crameri M Bauer M Caduff N Walker R Steiner F Franzoso FD. MxB is an interferon-induced restriction factor of human herpesviruses. Nat Commun. (2018) 9:1980. 10.1038/s41467-018-04379-2 29773792 PMC5958057

[152] Schilling M Bulli L Weigang S Graf L Naumann S Patzina C. Human MxB protein is a pan-herpesvirus restriction factor. J Virol. (2018) 92:e01056–18. 10.1128/JVI.01056-18 29950411 PMC6096802

[153] Jaguva Vasudevan AA Bähr A Grothmann R Singer A Häussinger D Zimmermann A. MXB inhibits murine cytomegalovirus. Virology. (2018) 522:158–67. 10.1016/j.virol.2018.07.017 30032029

[154] Hayashi ML Blankenship C Shenk T. Human cytomegalovirus UL69 protein is required for efficient accumulation of infected cells in the G1 phase of the cell cycle. Proc Natl Acad Sci USA. (2000) 97:2692–6. 10.1073/pnas.050587597 10706637 PMC15991

[155] Aoyagi M Gaspar M Shenk TE. Human cytomegalovirus UL69 protein facilitates translation by associating with the mRNA cap-binding complex and excluding 4EBP1. Proc Natl Acad Sci USA. (2010) 107:2640–5. 10.1073/pnas.0914856107 20133758 PMC2823912

[156] Collins-McMillen D Stevenson EV Kim JH Lee BJ Cieply SJ Nogalski MT. Human cytomegalovirus utilizes a nontraditional signal transducer and activator of transcription 1 activation cascade via signaling through epidermal growth factor receptor and integrins to efficiently promote the motility, differentiation, and polarization of infected monocytes. J Virol. (2017) 91:e00622–17. 10.1128/JVI.00622-17 29021395 PMC5709601

[157] Ishii KJ Coban C Kato H Takahashi K Torii Y Takeshita F. A Toll-like receptor–independent antiviral response induced by double-stranded B-form DNA. Nat. Immunol. (2006) 7:40–8. 10.1038/ni1282 16286919

[158] Dixit E Boulant S Zhang Y Lee AS Odendall C Shum B. Peroxisomes are signaling platforms for antiviral innate immunity. Cell. (2010) 141:668–81. 10.1016/j.cell.2010.04.018 20451243 PMC3670185

[159] Castanier C Garcin D Vazquez A Arnoult D. Mitochondrial dynamics regulate the RIG-I-like receptor antiviral pathway. EMBO Rep. (2010) 11:133–8. 10.1038/embor.2009.258 20019757 PMC2828750

[160] Magalhães AC Ferreira AR Gomes S Vieira M Gouveia A Valença I. Peroxisomes are platforms for cytomegalovirus' evasion from the cellular immune response. Sci Rep. (2016) 6:26028. 10.1038/srep26028 27181750 PMC4867596

[161] Geist LJ Dai LY. Cytomegalovirus modulates interleukin-6 gene expression. Transplantation. (1996) 62:653–8. 10.1097/00007890-199609150-00020 8830832

[162] Zhong Z Wen Z Darnell JE Jr. Stat3: a STAT family member activated by tyrosine phosphorylation in response to epidermal growth factor and interleukin-6. Science. (1994) 264:95–8. 10.1126/science.8140422 8140422

[163] Reitsma JM Sato H Nevels M Terhune SS Paulus C. Human cytomegalovirus IE1 protein disrupts interleukin-6 signaling by sequestering STAT3 in the nucleus. (2013). J Virol. 87:10763–76. 10.1128/JVI.01197-13 23903834 PMC3807375

[164] Harwardt T Lukas S Zenger M Reitberger T Danzer D Übner T. Human cytomegalovirus immediate-early 1 protein rewires upstream STAT3 to downstream STAT1 signaling switching an IL6-type to an IFNγ-like response. PLoS Pathog. (2016) 12:e1005748. 10.1371/journal.ppat.1005748 27387064 PMC4936752

[165] Peltier DC Lazear HM Farmer JR Diamond MS Miller DJ. Neurotropic arboviruses induce interferon regulatory factor 3-mediated neuronal responses that are cytoprotective, interferon independent, and inhibited by Western equine encephalitis virus capsid. J Virol. (2013) 87:1821–33. 10.1128/JVI.02858-12 23192868 PMC3554193

[166] Becker J Kinast V Döring M Lipps C Duran V Spanier J. Human monocyte-derived macrophages inhibit HCMV spread independent of classical antiviral cytokines. Virulence. (2018) 9:1669–84. 10.1080/21505594.2018.1535785 30403913 PMC7000197

[167] Wu Z Qin R Wang L Bosso M Scherer M Stamminger T Human cytomegalovirus particles treated with specific antibodies induce intrinsic and adaptive but not innate immune responses. J Virol. (2017) 91:e00678–17. 10.1128/JVI.00678-17 28878085 PMC5660505

[168] Dos Santos PF Van Weyenbergh J Delgobo M Oliveira Patricio D Ferguson BJ Guabiraba R. ISG15-induced IL-10 is a novel anti-inflammatory myeloid axis disrupted during active tuberculosis. J Immunol. (2018) 200:1434–42. 10.4049/jimmunol.1701120 29311364

[169] Botto S Streblow DN DeFilippis V White L Kreklywich CN Smith PP. IL-6 in human cytomegalovirus secretome promotes angiogenesis and survival of endothelial cells through the stimulation of survivin. Blood. (2011) 117:352–61. 10.1182/blood-2010-06-291245 20930069 PMC3037756

[170] Humar A. St. Louis P Mazzulli T McGeer A Lipton J Messner H. Elevated serum cytokines are associated with cytomegalovirus infection and disease in bone marrow transplant recipients. J Infect Dis. (1999) 179:484–8. 10.1086/314602 9878035

[171] Limaye AP Stapleton RD Peng L Gunn SR Kimball LE Hyzy R Effect of ganciclovir on IL-6 levels among cytomegalovirus-seropositive adults with critical illness: a randomized clinical trialganciclovir and IL-6 levels among CMV-seropositive critically Ill adultsganciclovir and IL-6 levels among CMV-seropositive critically Ill adults. JAMA. (2017) 318:731–40. 10.1001/jama.2017.10569 28829877 PMC5817487

[172] Bayer C Varani S Wang L Walther P Zhou S Straschewski S. Human cytomegalovirus infection of M1 and M2 macrophages triggers inflammation and autologous T-cell proliferation. J Virol. (2013) 87:67–79. 10.1128/JVI.01585-12 23055571 PMC3536399

[173] DeFilippis V Fruh K. Rhesus cytomegalovirus particles prevent activation of interferon regulatory factor 3. J Virol. (2005) 79:6419–31. 10.1128/JVI.79.10.6419-6431.2005 15858025 PMC1091669

